# 2D Synthetic dataset of numerical simulations of long-period seismicity in a volcanic edifice and related sensitivity kernels^☆^

**DOI:** 10.1016/j.dib.2020.106673

**Published:** 2021-01-25

**Authors:** Beatriz Martínez Montesinos, Christopher J. Bean, Ivan Lokmer

**Affiliations:** a School Of Earth Sciences, University College Dublin, Dublin 4, Ireland; b Geophysics Section, School of Cosmic Physics, Dublin Institute for Advanced Studies, Dublin 2, Ireland; c Istituto Nazionale di Geofisica e Vulcanologia, Sezione di Bologna, Bologna, Italy

**Keywords:** Numerical simulations, Volcanic edifice, Near surface velocity structure, Volcano seismic sources, Sensitivity kernels, Specfem2d

## Abstract

This work describes the data used in the EPSL research article “Quantifying strong seismic propagation effects in the upper volcanic edifice using sensitivity kernels”. The dataset is generated in order to investigate to what extent the seismic signals recorded on volcanoes are affected by near surface velocity structure. Data were calculated using the computational spectral elements scheme SPECFEM2D, where the wave propagation beneath Mount Etna volcano, Italy, was simulated in both homogeneous and heterogeneous models. The heterogeneous model comprises a low-velocity superficial structure (top several hundred meters) based on the previously published studies. Several different source mechanisms and locations were used in the simulations. The seismic wavefield was “recorded” by 15 surface receivers distributed along the surface of the volcano. The associated sensitivity kernels were also computed. These kernels highlight the region of the velocity model that affects the recorded seismogram within a desired time window. The text files describing the velocity models used in the simulations are also provided. The data may be of interest to volcano seismologists, as well as earthquake seismologists studying path effects and wave propagation through complex media.

## Specifications Table

**Table:** 

Subject	Earth and Planetary Sciences, Geophysics
Specific subject area	Volcano seismology
Type of data	ASCII files:
	- velocity models
	- synthetic seismograms
	- sensitivity kernels
How data were acquired	Data were calculated by performing numerical simulations of wave propagation in 2D models of Mt Etna, Italy, using the software package specfem2d ([1]).
Data format	Synthetic data (ASCII)
Parameters for data collection	(a) Two 2D velocity models of Mt Etna, Italy:
	- Homogeneous model created by considering P- and S-wave velocities and density Vp=3500 m/s, Vs=2384 m/s and ρ=2000 $kg/m^{3},$ respectively,
	- Heterogeneous model generated from Table 1 below,
	(b) Digital topography obtained from the CGIAR consortium for spatial information described in file *topography.txt*,
	(c) Different source and receiver locations.
Description of data collection	For every model, simulations of seismic wave propagation have been run using as seismic sources a single vertical force or an explosion with frequencies of 0.7 or 1 Hz located at 113 or 3613 m depth (relative positions (9000 m, 3000 m) for the shallow one and (9000 m, −5000 m) for the deeper one). Information on seismic sources is summarized in Tables 2 and 3 and in files *source_location.txt* and *source_type.txt*. Signals have been collected at 15 recording stations distributed along the surface with a station spacing of 1000 m whose relative positions are defined by Table 4 and in file *stations.txt*. Finally, associated sensitivity kernels were calculated for every 3 seconds time slice.
Data source location	The data were generated at the Irish Centre for High End Computing (ICHEC)
Data accessibility	With the article
Related research article	Beatriz Martínez Montesinos, Christopher J. Bean, Ivan Lokmer
	Quantifying strong seismic propagation effects in the upper volcanic edifice using sensitivity kernels
	Earth and Planetary Sciences Letters
	https://doi.org/10.1016/j.epsl.2020.116683

## Value of the Data

• This synthetic dataset highlights strong path effects on the long-period waveforms due to the shallow structure beneath volcanoes, supporting previous studies showing that, even if long-period seismicity are often associated with magma movements in resonant conduits (e.g: [2]), the corresponding wavefield can be significantly distorted by near-surface structure and surface topography (e.g. [3], [4]),
• The data are useful for volcano seismologists, and seismologists in general studying path effects and wave propagation through complex media,
• The data could be used for the investigation of seismic inversions in the presence of low velocity layering,
• This type of data usually requires quite a long time to calculate (including setting up the models). Therefore, it may help provide data to communities who do not have easy access to or experience with numerical wave simulation tools.

## 1 Data Description

The presented dataset has been calculated with the aim of investigating the highly complex nature of seismic wave propagation in near surface volcanic structures and demonstrate the extent to which resonating LP events can be caused by the structural model of the volcanic edifice. Files can be divided into five groups. The first one contains the input parameters that have been used to run the SPECFEM2D simulations. The second and third groups contain the velocity models and the source-time functions generated by SPECFEM2D based on the material properties and the source characteristics, respectively, defined in the first group. The last two groups contain the seismograms and the sensitivity kernels resulting from the simulations.

• Input parameters (Folder *SIMULATIONS_INPUT*)
  1. *material_properties.txt*: This file contains four columns with information about the seismic properties of each of the layers in the 2D model as in Table 1. The first column indicates the thickness of the layer, and the second, third and fourth columns give the velocities of the P- and S- waves and the density, respectively.

**Table 1:** Seismic velocities and density structure for the heterogeneous model. The S-wave velocities for the first 360 m depth are from [5] reporting the S-wave velocity structure derived from probabilistic inversion of Rayleigh-wave dispersion data. Deeper velocities come from [6]. Corresponding P-wave velocities are obtained using a Poisson’s ratio of 0.25 and densities using the Gardner’s relationship ([7]).

Thickness (m)	$v_{p}\left(m/s\right)$	$v_{s}\left(m/s\right)$	$\rho (kg/m^{3})$
60	745	430	1620
60	1334	770	1873
60	1957	1130	2062
120	2304	1330	2148
60	2771	1600	2249
1640	3250	1876	2341
4255	4800	2771	2580
  2. *topograpy.txt*: Contains two columns with the horizontal and vertical coordinates in meters of the 2D model’s surface of Mt Etna obtained from the CGIAR consortium for spatial information.
  3. *stations.txt*: Contains two columns with the horizontal and vertical position of the 15 recording stations on the 2D model as in Table 4.
  4. *source_location.txt*: Contains three columns with the depth and the horizontal and vertical coordinates in meters on the 2D model of the two seismic sources used in the simulations as in Table 2.

**Table 2:** Seismic source locations used for the simulations.

Depth (m)	Horizontal position (m)	Vertical position (m)
113	9000	3000
3613	9000	−500
  5. *source_type.txt*: Contains two columns indicating the frequencies in Hertz and mechanisms of the seismic sources used on the simulations as in table Table 3.

**Table 3:** Source frequency and mechanism used for the simulations.

Frequency (Hz)	Mechanism
0.7	Single vertical force (SVF)
0.7	Explosion (ISO)
1	Single vertical force (SVF)
1	Explosion (ISO)

**Table 4:** Seismic recording stations used for the simulations.

Horizontal position (m)	Vertical position (m)
3000	1551
4000	1655
5000	1768
6000	1904
7000	2202
8000	2685
9000	3113
10000	3210
11000	2830
12000	2493
13000	2105
14000	1946
15000	1795
16000	1580
17000	1320
• Velocity models created by SPECFEM2D based on input material properties (Folder *OUTPUT_VELOCITY_MODELS*) describing seismic velocities and density at every point of the 2D model of Mt Etna considered in the simulations. Every file contains 6 columns giving the identifier of the grid point, its relative horizontal and vertical position in meters, the P-wave and the S-wave velocities in m/s and the density in $kg/m^{3}$.
  1. *homo_velocity_model.txt* corresponds to the homogeneous model created by considering P- and S-wave velocities 3500 m/s and 2384 m/s, respectively and density 2000 $kg/m^{3}$.
  2. *hetero_velocity_model.txt* corresponds to the heterogeneous model created by considering seismic velocities and densities defined in Table 1 or file *material_properties.txt*.
• Source-time functions created by SPECFEM2D based on input source type (Folder *SOURCE_ TIME_FUNCTIONS*) describing the source-time history of the seismic source considered in the simulations. In the files, the first column indicates the time in seconds and second column the amplitude of the seismic source at this moment.
  1. *source_7e-1Hz.txt* corresponds to the 0.7 Hz source.
  2. *source_1e0Hz.txt* corresponds to the 1 Hz source.
• Output seismograms (Folder *OUTPUT_SEISMOGRAMS*) collected at the recording stations (Table 4 or file *stations.txt*) giving the value of displacements or velocities through time. Each of the files corresponds to a particular recording station and has been generated from a specific velocity model and seismic source. Every file contains two columns, the first one contains the time in seconds and the second one contains the displacement in μm for the case of displacement seismograms or the velocity in μm/s for the velocity seismograms. Files are stored and named indicating the parameters used to perform the simulation. For example:
  *OUTPUT_SEISMOGRAMS/9000_-500 /disp_X_homo_3000_1551_SVF7e-1Hz.semd* corresponds to the horizontal component of the displacement seismogram collected by the station located at (3000,1551) from a simulation using the homogeneous velocity model and the 0.7 Hz seismic source applied as a single vertical force at position (9000,-500),
  *OUTPUT_SEISMOGRAMS/9000_-3000/vel_Z_hetero_14000_1946_ISO1e0Hz.semv* corresponds to the vertical component of the velocity seismogram collected by the station located at (14000,1946) from a simulation using the heterogeneous velocity model and the 1 Hz seismic source applied as an explosion at position (9000,3000).
• Output sensitivity kernels (Folder *OUTPUT_KERNELS*) describing the region of the velocity model that affects the wavefield which is arriving at a given station within a defined time window. Given a seismogram, sensitivity kernels are provided for every 3 s time window. Files contain five columns. The first two columns contain the horizontal and vertical coordinates in meters of each point in the 2D model and the next three columns contain the density, P-wave and S-wave sensitivity kernels, respectively. Files are stored and named indicating the parameters used to perform the simulation and the time window for which the kernels are calculated. For example:
  *OUTPUT_KERNELS/9000_3000 /kernel_homo_3000_1551_SVF7e-1Hz_03-06.dat* is the kernel corresponding to the time window (3,6) seconds of the seismogram collected by the station located at (3000,1551) from a simulation using the homogeneous velocity model and the 0.7 Hz seismic source applied as a single vertical force at position (9000,3000),

## 2 Experimental Design, Materials and Methods

The spectral elements based software SPECFEM2D ([1]) was employed to perform 2D numerical simulations of seismic wave propagation in 2D models of Mount Etna, Italy, resulting in synthetic displacement and velocity seismograms. In addition to these seismograms, associated travel time sensitivity kernels were calculated using the adjoint methodology presented in [8] and implemented as well in SPECFEM2D. The seismograms were calculated for a selected source-receiver pair and the associated kernels were calculated for every three seconds time windows of the seismograms. These kernels show the areas of the volcano that mostly affect the wavefield arriving at the seismic stations within the seismogram time window under investigation. The calculation procedure can be described in four steps:

1. Creation of the two 2D models of Mount Etna, Italy. The digital topography was obtained from the CGIAR consortium for spatial information (*topograpy.txt*). Two velocity models were used in the calculation: homogeneous and heterogeneous. For the homogeneous model seismic velocities Vp=3500 m/s and Vs=2384 m/s and density=2000 kg/m3 were used. For the heterogeneous one S-wave velocities were extracted from [5] for the first 360 m depth and deeper velocities from [6] (*material_properties.txt*). Since the Poissons ratio is not well constrained (e.g. [9], [10], [11], [12], [13]), the typical value for the Earth’s crust of 0.25 was used to calculate the corresponding P wave velocities. Although it may affect details of the calculated seismograms, such a choice did not affect the core findings of this work. The densities were calculated using the Gardner relationship ([7])
  (1) $$\rho =310{v_{p}}^{0.25},$$
  where $v_{p}$ is measured in m s${}^{-1}$ and density in kg m${}^{-3}$.
2. Definition of the seismic sources. Since the objective of this work is to study low frequency volcanic seismicity and structure tuning effects, the data were calculated for two different source depths, two different source mechanisms and two different source-time functions (*source_location.txt, source_type.txt*). The shallow source was located at 113 m deep (within the low velocity superficial layer) and the deep one at 3613 m deep. The source was a Ricker wavelets applied either as a single vertical force or as an explosion. We also considered two different source central frequencies, 0.7 Hz and 1 Hz. Such closely spaced frequencies were chosen to test sensitivity to small central frequency differences in the most common range of long period (LP) frequencies.
3. Definition of the seismic network. Seismic network contained 15 stations along the volcano surface with the interstation spacing of 1000 m (*stations.txt*).
4. Running simulations. Utilizing the 2D models, the sources and the stations described previously, systematic forward and adjoint simulations of wave propagation were performed by SPECFEM2D for different combinations of source locations, mechanisms, frequencies and velocity models. Sensitivity kernels were calculated using the same software package. These simulations resulted in the seismograms collected by each of the seismic stations (folder *OUTPUT_SEISMOGRAMS*) and the sensitivity kernels for each 3 seconds time windows of the investigated time window. These kernels highlight the regions in the subsurface velocity models which have the strongest effect on each time segments in the seismogram (folder *OUTPUT_KERNELS*). Meshed velocity models detailed for each point of the 2D model structure (*homo_velocity_model.txt* and *hetero_velocity_model.txt*) and the source time function corresponding to each of the two frequency sources (*source_7e-1Hz.txt* and *source_1e0Hz.txt*) were also provided.

## Declaration of Competing Interest

The authors declare that they have no known competing financial interests or personal relationships which have, or could be perceived to have, influenced the work reported in this article.

## Appendix A Supplementary materials

Supplementary Data S1 Supplementary Raw Research Data. This is open data under the CC BY license http://creativecommons.org/licenses/by/4.0/ Supplementary Data S1
