# Supplementary material for: 2D Synthetic dataset of numerical simulations of long-period seismicity in a volcanic edifice and related sensitivity kernels
Source: Data Brief. 2021 Jan 25;34:106673. doi: 10.1016/j.dib.2020.106673 (PMC7841316; doi:10.1016/j.dib.2020.106673)
Supplement: Supplementary Data S1 — Supplementary Raw Research Data. This is open data under the CC BY license http://creativecommons.org/licenses/by/4.0/ [file mmc1.zip › Dataset/SOURCE_TIME_FUNCTIONS/README.rtf]

The simulations have been carried out with an amplification factor of 0.75d6
